# Correlation between Parental Transcriptome and Field Data for the Characterization of Heterosis in Chinese Cabbage

**DOI:** 10.3390/genes14040776

**Published:** 2023-03-23

**Authors:** Ru Li, Min Tian, Qiong He, Lugang Zhang

**Affiliations:** State Key Laboratory of Crop Stress Biology for Arid Area, College of Horticulture, Northwest A&F University, Yangling 712100, China

**Keywords:** heterosis, differential expression genes, Euclidean distance, binary distance

## Abstract

In Chinese cabbage breeding, hybrids have made a terrific contribution due to heterosis, the superior performance of offspring compared to their inbred parents. Since the development of new, top-performing hybrids requires a large scale of human and material resources, the prediction of hybrid performance is of utmost interest to plant breeders. In our research, leaf transcriptome data from eight parents were used to investigate if they might be employed as markers to predict hybrid performance and heterosis. In Chinese cabbage, heterosis of plant growth weight (PGW) and heterosis of head weight (HW) were more obvious than other traits. The number of differential expression genes (DEGs) between parents was related to the PGW, length of the biggest outer leaf (LOL), leaf head height (LHH), leaf head width (LHW), HW, leaf number of head (LNH) and plant height (PH) of hybrids, and up-regulated DEGs number was also associated with these traits. Euclidean and binary distances of parental gene expression levels were significantly correlated with the PGW, LOL, LHH, LHW, HW and PH of hybrids. Additionally, there was a significant correlation between the parental expression levels of multiple genes involved in the ribosomal metabolic pathway and hybrid observations and heterosis in PGW, with the *BrRPL23A* gene showing the highest correlation with the MPH of PGW(r = 0.75). Therefore, leaf transcriptome data can preliminarily predict the hybrid performance and select parents in Chinese cabbage.

## 1. Introduction

Heterosis is a phenomenon in which hybrids outperform their homozygous parents in vitality, growth vigor, fecundity, yield, quality, stress resistance and adaptability [1,2,3,4,5]. As a common biological phenomenon, heterosis can be observed in almost all sexually reproducing species, from plants to animals, and even in microorganisms. Firstly, Darwin systematically studied heterosis as a result of the hybridization of organisms with different genetic components and further proposed that heterozygous pollination is beneficial to plants while self-pollination is detrimental. Then, the dominant hypothesis [6], over-dominance [7,8,9], epistatic effects [10,11] and other hypotheses were proposed to explain the formation mechanism of heterosis. Since its introduction, heterosis has emerged as the primary method for increasing the yield of grain [12,13], oil crops [14] cotton [15,16] and vegetables [17,18]. Meanwhile, the use of heterosis can also improve the stress resistance and adaptability of crops. Heterosis has contributed significantly to global food production, brought about enormous economic and social advantages, and is also a prominent achievement of modern agricultural biotechnology [19,20].

The identification of new superior hybrids among a large number of possible crosses in new parental lines generated each year requires extensive testing programs, including the production of numerous test crosses, extensive multi-location/-year field trials to generate phenotypic data and to test hybrid performance [21]. Therefore, using data collected from parental inbred lines to predict the performance of hybrids promises to improve the efficiency of cross-breeding and is of great interest to breeders. Currently, field data, DNA markers, whole-genome data, transcriptome data and so on, are used to predict the performance of hybrids and to analyze the relationship between various characteristics of parents and heterosis in hybrids for improving breeding efficiency [22,23,24].

With further research on heterosis, genetic distance has been used as a measure of the degree of genetic differences between parents to select parents and predict hybrid performance. In general, genetic differences between parents are greater and hybrid offspring have the more obvious hybrid heterosis, but this does not imply that this relationship will be across the whole range of species diversity [25,26,27]. The hump quadratic polynomial function was found between the genetic distance of parents and the phenotype of hybrids. Within a certain range, heterosis raises with increasing genetic distance between parents, but beyond this range, heterosis tends to decrease with increasing genetic distance [28]. In addition, some studies indicated that heterosis was significantly correlated with the genetic distance between parents [29,30,31] or had no obvious relationship [32]. Investigating the relationship between parental genetic distance and hybrid phenotypic traits is an essential combination of molecular genetics and conventional breeding [33]. Therefore, the study on the relationship between parental genetic distance and heterosis is crucial for the effective prediction of heterosis, scientific guidance on parental selection and rational use of heterosis.

Chinese cabbage (*Brassica rapa* L. ssp. *pekinensis*), which originated in China, is one of the largest and most productive vegetable crops grown in China. It is highly consumed in Asian countries and is one of the most important vegetable crops in the world [34,35,36]. In Chinese cabbage, heterosis is evident and employed as an effective way and important means to improve yield, disease resistance, stress resistance and quality. However, there are few studies about predicting the performance of Chinese cabbage hybrids. Thus, there is a need to find a method for selecting parents and predicting the performance of hybrids in Chinese cabbage.

In this study, eight inbred lines and 53 hybrids were used as plant materials to explore whether parental transcriptome data could be used to predict hybrid performance and select parents. Firstly, the correlation between the parental number of DEGs and hybrid performance was calculated. Secondly, the correlation between the parental genetic distance based on transcriptome data and hybrid performance was counted. Finally, the gene-related heterosis was identified by analyzing the correlation between parental expression level and the performance of hybrids. These analyses are conducive to the application of transcriptome data in heterosis prediction, and also provide a reference for heterosis prediction in breeding process of Chinese cabbage.

## 2. Materials and Methods

### 2.1. Plant Materials

Eight inbred lines and 53 hybrids were used for heterosis analysis (Table 1). All 8 Chinese cabbage inbred lines were developed and provided by the Chinese cabbage research group, at the College of Horticulture, Northwest A&F University, Yangling, China, which were self-bred for at least eight generations. Appling complete diallel crossing design, the inbred line parents of Chinese cabbage were used for artificial cross-pollination to obtain the hybrids. The details of the crosses were presented in Table 1. In all the materials, inbred lines A, B, C, D, E, F, G, and H were parents, and the other materials were hybrids.

The parents and hybrids were cultured in the same experimental field at the Yangling Wuquan test field in Shaanxi, China. At the middle heading stage (about 70 days), the first outer leaf of the Chinese cabbage parents was collected from top to bottom as an RNA-Sep sample. Three individual plants were mixed as a test sample, and three replicates were selected for each material. At the maturity stage(about 100 days), parents and hybrids were investigated for yield traits and yield related traits including plant growth weight(PGW)(data from our previous project) [37], head weight(HW), plant width (PW), PH, number of outer leaves(NOL), LOL, width of the biggest outer leaf(WOL), leaf head height(LHH), leaf head width(LHW), and leaf number of head(LNH).

### 2.2. Heterosis Statistical Analysis

Data collected from the field were used to analyze the heterosis of traits. The heterosis require the calculation of mid-parent heterosis (MPH) and high-parent heterosis (HPH). The formulae for their calculation are as follows:

(1) $$\mathrm{MPH}\;=\frac{F1-\mathrm{MP}}{\mathrm{MP}}\times 100\%$$

(2) $$\mathrm{HPH}\;=\frac{F1-\mathrm{HP}}{\mathrm{HP}}\times 100\%$$

where F_1_ is the value of hybrid, MP is the mean value of two parents, and HP is the value of the better parent.

### 2.3. RNA Extraction, Library Construction and RNA-Seq

Total RNA was extracted using the Trizol reagent following the manufacturer’s instructions by Genedenovo Biotechnology Co., Ltd. (Guangzhou, China). The RNA quality and concentration were examined using an Agilent 2100 Bioanalyzer (Agilent Technologies, Santa Clara, CA, USA). The mRNA was isolated using magnetic beads with Oligo (dT) and fragmented into small pieces using fragmentation buffer. Then, the mRNA fragments were used as templates to synthesize the first strand of cDNA with random hex base random primers and the second chain of cDNA with buffer, dNTPs, RNase H and DNA polymerase I. The synthesized cDNA were purified using a QiaQuick PCR extraction kit and subjected to end reparation and single nucleotide A (adenine) addition. Thereafter, the short fragments were ligated to Illumina sequencing adapters and the suitable sized fragments were selected as templates for PCR amplification. Finally, the transcriptome libraries were sequenced using Illumina HiSeq™2500 by Genedenovo Biotechnology Co., Ltd. (Guangzhou, China). The obtained raw data from constructed cDNA libraries were deposited in NCBI Sequence Read Archive (SRA, http://www.ncbi.nlm.nih.gov/Traces/sra/ accessed on 1 June 2022) under the accessionnumber BioProject PRJNA876066 [37].

### 2.4. Differentially Expressed Genes Analysis

Raw reads from RNA-seq were obtained from our previous project [37], and then mapped to the Chinese cabbage genome sequences from the Brassica database (http://brassicadb.org/brad) using TopHat2 software [38]. Gene expression levels were normalized using the fragments per kilobase of transcript sequence per millions (FPKM) method. Differentially expressed genes between groups were analyzed using Edge software. The FDR < 0.05 and |log2^FC^| > 1 were used as the threshold to identify significant DEGs.

### 2.5. Transcriptome-Based Distance Analysis

Euclidean and binary distances were employed as indicators to measure the parental differences and were calculated by parental transcriptome data. The expression level of all genes was calculated, and then Euclidean and binary distances between parents were calculated based on the R language.

### 2.6. Identifying Genes Correlated to PGW and MPH

The mean, maximum and minimum valuess of parental gene expression level were calculated as mid-parent expression val high-parent expressionion value, and low-parent expression value, respectively. Correlation coefficients of high-parent expression value, mid-parent expression value, and low-parent expression value with measured value and MPH of PGW in hybrids were calculated. Pearson’s product–moment correlation in R was used to test the significance of the correlation coefficients. For multiple testing corrections, *p* values were adjusted with a false discovery rate of 0.01.

### 2.7. GO and KEGG Enrichment Analysis

To identify possible biological functions of DEGs, Gene Ontology (GO) and Kyoto Encyclopedia of Genes and Genomes (KEGG) enrichment analyses were performed. The DEGs were mapped to terms in the GO database (http://www.geneontology.org/). Then, significantly enriched GO terms were searched by comparing them to the genome background with an adjusted *p*-value ≤ 0.05 as the threshold. KOBAS software was used for pathway enrichment analysis based on the KEGG database [39]. A corrected *p* value ≤ 0.05 was used as the threshold to identify the significantly enriched functional terms and pathways.

## 3. Results

### 3.1. Statistical Analysis of 10 Traits in Hybrids and Parents of Chinese Cabbage

By analyzing the traits of parents and hybrids, it was found that the hybrids had higher means in these traits, including PGW, NOL, LOL, WOL, LHH, LHW, HW, LNH, PW and PH, compared to the parents (Table 2). For instance, the mean of hybrids is 3.75 kg, ranging from 1.40 kg to 6.50 kg, whereas the mean of the parents for PGW is 2.13 kg, ranging from 0.53 kg to 3.53 kg. The PGW mean in hybrids was higher than that in the parents, and other traits followed a similar pattern to the PGW.

### 3.2. Heterosis of 10 Traits in Hybrids of Chinese Cabbage

**Figure 1:**
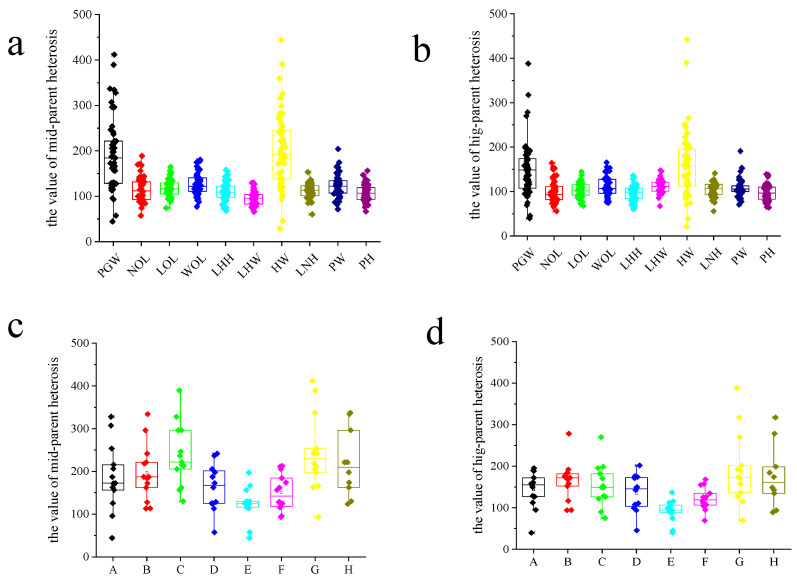
The mid-parent heterosis value and high-parent heterosis value in hybrids. (**a**) The mid-parent heterosis value of different traits in 53 hybrids. (**b**) The high-parent heterosis value of different traits in 53 hybrids. (**c**) The mid-parent heterosis value of plant growth weight with different parents. (**d**) The high-parent heterosis value of plant growth weight with different parents. PGWplant growthth weight, NOL: number of outer leaves, LOL: length of the biggest outer leaf, WOL: width of the biggest outer leaf, LHH: leaf head height, LHW: leaf head width, HW: head weight, LNH: leaf number of head, PW: plant width, PH: plant height. A, B, C, D, E, F, G and H were parent inbred lines.

Among the 53 hybrids, the mean MPH was higher for PGW and HW compared to other traits (Figure 1a). The MPH for PGW was 192.54, ranging from 44.49 to 411.90, while the MPH for HW was 201.78, with a range of 28.40 to 444.02. The average MPH of NOL, LOL, WOL, LHH, LHW, LNH, PW and PH were 115.03, 117.62, 127.10, 109.58, 95.17, 112.08, 122.90 and 106.83, respectively. For these traits, HPH had a similar pattern to MPH. Compared to other traits, PGW and HW had higher average HPH (Figure 1b). The average HPH for PGW and HW were 149.49 and 160.37, respectively. The average HPH for NOL, LOL, WOL, LHH, LHW, LNH, PW and PH was 98.64, 103.38, 111.10, 96.39, 111.53, 103.50, 108.43 and 96.89, respectively.

The heterosis of hybrids varied among parents. For PGW, the average MPH of hybrids was higher than that of hybrids crossed with other parents when the inbred lines C, G and H were used as parents (Figure 1c). When inbred lines C, G and H were used as parents, the averages MPH of hybrids were 249.37, 247.20 and 219.78, respectively. When inbred lines E and F were used as parents, the average MPH of hybrids was lower than that of hybrids from other parental crosses, with the averages of 122.61 and 150.73. Among the eight parents, the hybrids from parents G and H had higher HPH, with the average of 196.41 and 176.16 (Figure 1d). The hybrids from parents E and F had lower HPH compared to other parents with mean values of 92.54 and 121.27, respectively. Overall, compared with other materials, parent G and H’s hybrids had more obvious heterosis, while parent E and F’ s hybrids had lower heterosis in PGW.

### 3.3. Correlation between the Parental DEGs Number and Hybrid Heterosis

To investigate the parental influence on heterosis, the correlations between the number of parental DEGs and heterosis of different traits in hybrids were analyzed. The results revealed that there was a significant correlation between the number of parental DEGs and the MPH of LNH (r = 0.30), but there was no significant correlation between the number of parental DEGs and the MPH of other traits (Table 3). The number of up-regulated DEGs (female parent vs. male parent) was only significantly correlated with the MPH of LNH (r = 0.51).

Analysis of the correlation between parental DEGs numbers and hybrid traits revealed that parental DEGs number was significantly correlated with several traits, including PGW, LOL, LHH, LHW, HW, LNH and PH (Table 4). The number of up-regulated DEGs (female parent vs. male parent) was significantly related to the observed value of PGW, LOL, WOL, LHH, LHW, HW, LNH and PH. However, the number of down-regulated genes (female parent vs. male parent) was not correlated with the observed value of all traits.

### 3.4. Correlation between the Transcriptome-Based Distances and Heterosis with Traits

To explain the effect of parental differences on hybrid heterosis, the correlation between parental genetic distance and hybrid heterosis for different traits was analyzed. The results suggested that the Euclidean distance between parents was significantly correlated with the hybrid MPH of PGW, LHH, HW, LNH and PH. The binary distance between parents was only significantly correlated with the MPH of LNH, but not with other traits (Table 5).

The Euclidean distance between parents was significantly correlated with the observed value of PGW, LOL, LHH, LHW, HW, PW and PH. The binary distance between parents was significantly correlated to observed value of PGW, LOL, LHH, LHW, HW, LNH and PH (Table 6). The correlation coefficients of traits with Euclidean distances were larger than binary distances, except for LNH.

### 3.5. Genes Correlated to PGW and MPH

**Figure 2:**
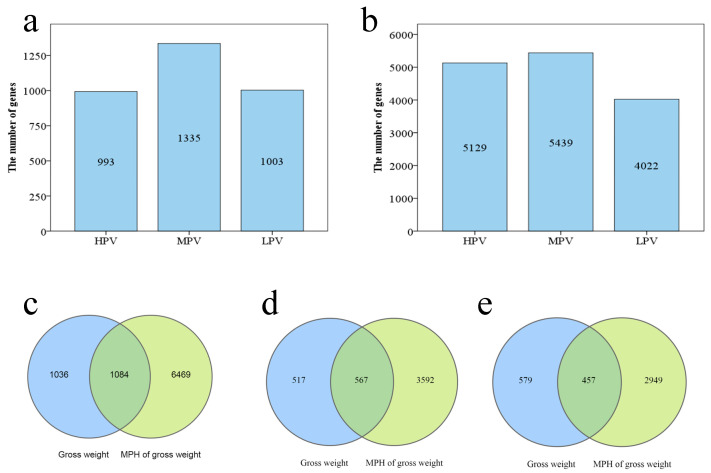
Number of genes correlated to observed value and mid-parent heterosis value of plant growth weight. (**a**) Number of genes correlated to plant growth weight. (**b**) Number of genes correlated to mid-parent heterosis value of plant growth weight. (**c**) Number of genes correlated to observed value and mid-parent heterosis value of plant growth weight. (**d**) Number of genes positively correlated to observed value and mid-parent heterosis value of plant growth weight. (**e**) Number of genes negatively correlated to observed value and mid-parent heterosis value of plant growth weight. HPV: high-parent expression. MPV: mid-parent heterosis value: mid-parent expression. LPV: low-parent expressionn.

Genes associated with PGW were identified by analyzing the correlation between parental gene expression levels and PGW in hybrids. In a total of 993 genes, 1335 genes and 1003 genes, the high-parent expression, mid-parent expression and low-parent expression were significantly correlated with the PGW in hybrids (Figure 2a). In addition, the correlation between the parental gene expression level and the MPH of PGW was analyzed. There were 5126 genes, 5439 genes and 4022 genes, in which the high-parent expression, mid-parent expression and low-parent expression were significantly correlated with the MPH of PGW of the hybrids, respectively (Figure 2b).

Among these genes, 1084 genes were significantly associated with the observed value and MPH of PGW (Figure 2c). Among these genes, 567 genes were significantly positively correlated with the measurements and MPH of PGW (Figure 2d), and 457 genes were negatively related to both the measured value and the MPH of PGW (Figure 2e).

### 3.6. Enrichment Analysis of Genes Related to Heterosis

**Figure 3:**
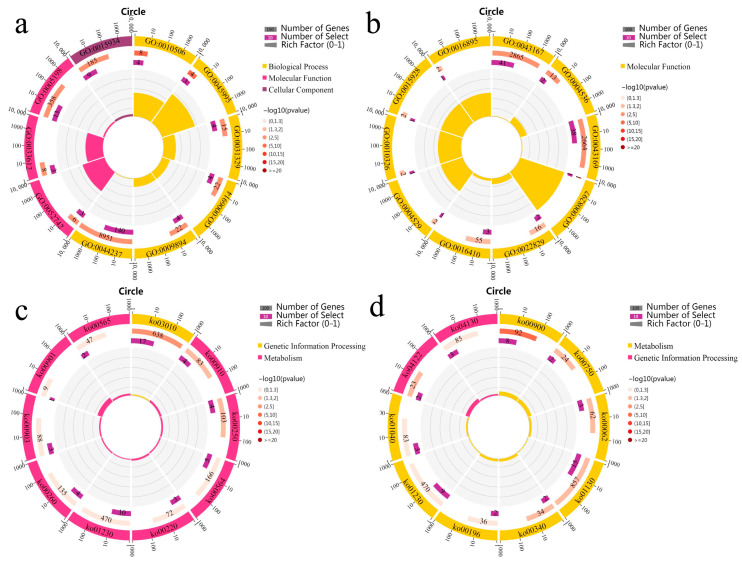
Enrichment analysis of genes correlated to plant growth weight. (**a**) GO enrichment analysis of genes positively correlated to observed value and mid-parent heterosis value of plant growth weight in hybrids. (**b**) GO enrichment analysis of genes negatively correlated to observed value and mid-parent heterosis valueof plant growth weight in hybrids. (**c**) KEGG enrichment analysis of genes positively correlated to observed value and mid-parent heterosis value of plant growth weight in hybrids. (**d**) KEGG enrichment analysis of genes negatively correlated to observed value and mid-parent heterosis value of plant growth weight in hybrids.

GO enrichment analysis was used to determine the function of 567 genes whose parental gene expression levels were positively correlated with the hybrid observed value and MPH of PGW. In the biological process, these genes were significantly enriched in the regulation of autophagy (GO: 0010506), regulation of embryonic development (GO: 0045995) and regulation of cellular catabolic process (GO: 0031329) (Figure 3a). In the molecular functions, these genes were significantly enriched in phosphatidylinositol kinase activity (GO: 0052742), receptor serine/threonine kinase binding (GO: 0033612) and structural molecular activity (GO: 0005198). In the cellular components, these genes were significantly enriched in large ribosomal subunit (GO: 0015934). A total of 457 genes, whose parental gene expression levels were negatively related to hybrid observed value and MPH of PGW, were significantly enriched in the molecular functional classification, including ion binding (GO: 0043167), deoxyribonuclease activity (GO: 0004536) and cation binding (GO: 0043169) (Figure 3b).

KEGG pathway enrichment analysis indicated that the genes, whose parental gene expression levels were positively related to hybrid observed value and MPH of PGW, were significantly enriched in ribosome (ko03010), nitrogen metabolism (ko00910) and alanine, aspartate and glutamic acid metabolism (ko00250) (Figure 3c). The most dominant pathways were ribosome (ko03010). These genes, which parental gene expression levels were negatively related to hybrid observed value and MPH of PGW, were significantly enriched in terpenoid backbone biosynthesis (ko00900), sulfur relay system (ko04122), vitamin B6 metabolism (ko00750), fatty acid elongation (ko00062), biosynthesis of antibiotics (ko01130) and histidine metabolism (ko00340) (Figure 3d). The most dominant pathways were terpenoid backbone biosynthesis (ko00900).

### 3.7. Metabolic Pathway Related to Heterosis

In the ribosome metabolic pathway, the parental gene expression levels of 17 genes were correlated with hybrid PGW, with a correlation coefficient ranging from 0.28 to 0.43, and related to hybrid MPH of PGW, with a correlation coefficient ranging from 0.31 to 0.75. Among these genes, the parental gene expression levels of *BraA03g010340.3C* (*BrRPL10AC*) showed the highest correlation with hybrid PGW (r = 0.43), and the parental gene expression levels of *BraA03g020910.3C* (*BrRPL23A*) had the highest correlation with hybrid MPH of PGW (r = 0.73) (Table 7).

In the terpenoid backbone biosynthesis metabolic pathway, eight genes had a significant negative correlation between gene expression levels in the parents and hybrid PGW with correlation coefficients ranging from −0.42 to −0.34 (Table 8). Among them, the parental expression level of *BraA01g044250.3C (BrIPP2)* had the highest correlation with hybrid PGW. The parental expression level of these genes was also significantly and negatively related to the MPH of PGW in hybrids, with a correlation coefficient ranging from −0.57 to −0.36. Among them, the parental expression level of *BraA08g025620.3C* (*BrICMEL1)* showed the highest correlation with the MPH of PGW.

## 4. Discussion

In Chinese cabbage, the selection of outstanding hybrids is also concentrated in the continual process of trying, which not only wastes a lot of manpower and material resources and causes economic waste but also severely hinders the use of heterosis. The key to using the heterosis is evaluating the parents of outstanding hybrids, but the evaluation process is time-consuming and labor-intensive and becomes the bottleneck of hybrid breeding. Despite this, it is not always possible to obtain strong heterosis hybrids by crossbreeding using excellent parents. To improve the breeding efficiency, previous efforts have attempted to develop a variety of methods for predicting heterosis, including combining the ability method, physiological and biochemical method and molecular marker method.

With the rapid development of various kinds of omics, the application of transcriptome data has emerged as a new approach for predicting heterosis. Frisch et al. discovered that the transcriptome-based distance was significantly correlated with the phenotype and heterosis in maize hybrids [23]. In maize, the proportion of genes with an additive expression pattern in all genes was significantly positively correlated with the phenotype and heterosis of hybrids [40]. These results indicated that transcriptome data can be used to predict heterosis. In this study, the association between the expression of the parental genes and the performance of hybrids was examined using transcriptome data. The results showed that the number of DEGs between parents was related to the field observation of PGW, LOL, LHH, LHW, HW, LNH and PH, and the number of up-regulated genes was related to these traits. In addition, Euclidean and binary distances of parental gene expression levels were significantly correlated with the PGW, LOL, LHH, LHW, HW and PH of hybrids. These results show that transcriptome data can preliminarily predict phenotype in Chinese cabbage hybrids. Therefore, the prediction of heterosis based on transcriptome has a significant potential to increase the effectiveness in a hybrid breeding program in some crops.

In hybrids, some parental gene expression levels were related to the performance of hybrids and could be used as predictors of hybrid performance. Thiemann et al. observed a significant correlation between gene expression levels in parents and traits and heterosis in hybrid. In Arabidopsis thaliana, a decrease in the abundance of *At3g11220* transcripts in the parents was significantly correlated with an increase in biomass heterosis in the corresponding region [24]. In maize, compared to other genes, the transcriptional abundance of AGAMOUS-like protein in parents showed the most significant correlation with hybrid traits [41]. The purpose of this study is to identify genes related to the PGW of hybrids by calculating the correlation among mid-parent expression level, high-parent expression level, low-parent expression level and PGW in hybrids. The results revealed that the expression level of multiple genes in the ribosome metabolism pathway was positively correlated with observation and the MPH of PGW, with the *BrRPL23A* gene showing the highest correlation with the MPH of PGW (r = 0.75), while the expression level of multiple genes in the terpenoid backbone biosynthesis metabolic pathway was negatively correlated with observation and MPH of PGW. Therefore, the parental expression levels of some genes were related to hybrid phenotypes, can preliminarily predict the heterosis and could provide reference for parents’ selection.

In the ribosome metabolism pathway, the expression level of *BrRPL23A* in parents had the highest correlation with heterosis of PGW. In Arabidopsis, *RPL23A* is a part of a generally conserved protein, located in the cytoplasm, and directly binds to large molecular subunit (LSU) RNA, which is necessary for ribosome biosynthesis [42]. *AtRPL23Aa* gene knockout can lead to plant growth retardation, leaf irregularity, leaf abscission, loss of root morphology and apical dominance, and the function of *RPL23A* is crucial for plant survival in Arabidopsis thaliana [43]. In conclusion, the parental expression level *BrRPL23* may related to the heterosis in Chinese cabbage and could be used to simply predict PGW of hybrids.

## 5. Conclusions

We concluded that parental DEGs number and transcriptome-based distance were related to hybrid phenotypes and could preliminarily predict hybrid phenotypes and select parents. In the ribosome metabolism pathway, the parental expression level of *BrRPL23* could be used to simply predict PGW of hybrids.

## Figures and Tables

**Table 1:** The codes of inbred lines and hybrids of Chinese cabbage.

	Male Parent	A	B	C	D	E	F	G	H
Female Parent									
A			AB	/	AD	AE	AF	AG	AH
B		BA		BC	BD	BE	BF	BG	BH
C		CA	CB		CD	CE	CF	CG	CH
D		DA	DB	DC		DE	DF	DG	DH
E		EA	EB	EC	ED		EF	EG	EH
F		FA	FB	FC	FD	FE		/	FH
G		/	GB	GC	GD	GE	GF		GH
H		HA	HB	HC	HD	HE	HF	HG	

The code in the first column represents the female parent, the code in the first line represents the male parent, and the rests are the corresponding hybrids. /: The material is missing.

**Table 2:** Analysis of phenotype variation of traits for parental inbred lines and hybrids.

Trait	Parents			Hybrids		
	Mean Value	Range of Variation	Variance Coefficient	Mean Value	Range of Variation	Variance Coefficient
PGW	2.13 ± 0.96	0.53–3.53	44.95	3.75 ± 0.99	1.40–6.50	26.33
NOL	8.63 ± 2.50	5.00–11.67	29.03	9.72 ± 2.05	6.33–17.00	21.13
LOL	38.36 ± 8.95	23.50–51.33	23.33	44.47 ± 5.59	32.67–56.00	12.57
WOL	24.40 ± 5.91	15.63–34.00	24.21	30.37 ± 3.76	22.00–37.50	12.39
LHH	27.25 ± 6.58	19.33–37.50	24.16	29.62 ± 5.13	19.00–42.14	17.32
LHW	17.76 ± 2.31	13.40–21.50	13.02	21.19 ± 2.29	13.00–26.50	10.83
HW	1.39 ± 0.65	0.46–2.49	46.59	2.57 ± 2.29	0.52–4.22	26.34
LNH	34.92 ± 4.82	27.67–41.33	13.81	39.14 ± 5.89	23.00–51.33	15.06
PW	54.63 ± 12.06	34.00–66.33	22.08	65.91 ± 8.32	45.50–93.67	12.62
PH	37.08 ± 6.91	29.33–49.00	18.64	39.25 ± 5.46	28.00–52.33	13.91

PGW: plant growth weight, NOL: number of outer leaves, LOL: length of the biggest outer leaf, WOL: width of the biggest outer leaf, LHH: leaf head height, LHW: leaf head width, HW: head weight, LNH: leaf number of head, PW: plant width, PH: plant height.

**Table 3:** The correlation between the number of parental differential expression genes and the mid-parent heterosis value of traits in hybrids.

Traits	The Number of Genes		
	UP	DOWN	ALL
PGW	0.091	0.054	0.096
NOL	−0.152	−0.134	−0.189
LOL	0.018	−0.076	−0.037
WOL	0.051	−0.109	−0.035
LHH	0.047	0.001	0.032
LHW	−0.121	−0.117	−0.157
HW	0.133	0.079	0.141
LNH	0.508 **	−0.074	0.298 *
PW	0	−0.014	−0.009
PH	0.155	−0.048	0.075

**: Correlation is significant at the 0.01 level, *: Correlation is significant at the 0.05 level. PGW: plant growth weight, NOL: number of outer leaves, LOL: length of the biggest outer leaf, WOL: width of the biggest outer leaf, LHH: leaf head height, LHW: leaf head width, HW: head weight, LNH: leaf number of head, PW: plant width, PH: plant height. UP: up-regulated DEGs (female parent vs. male parent), DOWN: down-regulated genes (female parent vs. male parent), ALL: DEGs (female parent vs. male parent).

**Table 4:** The correlation between the number of parental differentiall expression genes and observed value of traits in hybrids.

Traits	The Number of Genes		
	UP	DOWN	ALL
PGW	0.340 *	0.104	0.298 *
NOL	0.028	0.013	0.027
LOL	0.437 **	0.252	0.459 **
WOL	0.298 *	−0.017	0.192
LHH	0.350 *	0.254	0.401 **
LHW	0.327 *	0.234	0.372 **
HW	0.354 **	0.109	0.311 *
LNH	0.556 **	0.002	0.379 **
PW	0.255	0.132	0.259
PH	0.437 **	0.153	0.395 **

**: Correlation is significant at the 0.01 level, *: Correlation is significant at the 0.05 level. PGW: plant growth weight, NOL: number of outer leaves, LOL: length of the biggest outer leaf, WOL: width of the biggest outer leaf, LHH: leaf head height, LHW: leaf head width, HW: head weight, LNH: leaf number of head, PW: plant width, PH: plant height. UP: up-regulated DEGs (female parent vs. male parent), DOWN: down regulated genes (female parent vs. male parent), ALL: DEGs (female parent vs. male parent).

**Table 5:** Correlation between mid-parent heterosis value of traits and parents’ genetic distance.

	Binary	Euclidean
PGW	0.109	0.347 *
NOL	−0.191	0.082
LOL	−0.029	0.245
WOL	−0.025	0.207
LHH	0.041	0.449 **
LHW	−0.147	0.15
HW	0.154	0.398 **
LNH	0.298 *	0.337 *
PW	0.001	0.189
PH	0.081	0.301 *

**: Correlation is significant at the 0.01 level, *: Correlation is significant at the 0.05 level. PGW: plant growth weight, NOL: number of outer leaves, LOL: length of the biggest outer leaf, WOL: width of the biggest outer leaf, LHH: leaf head height, LHW: leaf head width, HW: head weight, LNH: leaf number of head, PW: plant width, PH: plant height.

**Table 6:** Correlation between observed value of traits and parents’ genetic distance.

	Binary	Euclidean
PGW	0.301 *	0.384 **
NOL	0.02	0.095
LOL	0.461 **	0.559 **
WOL	0.196	0.162
LHH	0.404 **	0.566 **
LHW	0.378 **	0.393 **
HW	0.316 *	0.394 **
LNH	0.379 **	0.263
PW	0.258	0.352 **
PH	0.399 **	0.401 **

**: Correlation is significant at the 0.01 level, *: Correlation is significant at the 0.05 level. PGW: plant growth weight, NOL: number of outer leaves, LOL: length of the biggest outer leaf, WOL: width of the biggest outer leaf, LHH: leaf head height, LHW: leaf head width, HW: head weight, LNH: leaf number of head, PW: plant width, PH: plant height.

**Table 7:** Correlation between parental gene expression levels in ribosome metabolic pathwayand plantgrowth weight in hybrids.

GeneID	Symbol	PGW		MPH of PGW	
		cor	Q Value	cor	Q Value
*BraA01g015640.3C*	*RPL7AB*	0.3184	0.0202	0.351	0.01
*BraA02g038190.3C*	*RPP2C*	0.3917	0.0037	0.378	0.0053
*BraA03g010340.3C*	*RPL10AC*	0.4304	0.0013	0.4291	0.0013
*BraA03g020910.3C*	*RPL23A*	0.318	0.0203	0.7465	1.39 × 10^−10^
*BraA03g047490.3C*	*RPL15A*	0.3514	0.0099	0.3096	0.0241
*BraA04g006490.3C*	*RPL24B*	0.3209	0.0191	0.4076	0.0025
*BraA04g014040.3C*	*RPS10B*	0.3328	0.0149	0.4193	0.0018
*BraA05g028570.3C*	*RPL30B*	0.366	0.007	0.4511	0.0007
*BraA06g029850.3C*	*RPL6*	0.4174	0.0019	0.4623	0.0005
*BraA06g032890.3C*		0.3173	0.0206	0.4261	0.0015
*BraA07g012190.3C*	*RPL17B*	0.3825	0.0047	0.3783	0.0052
*BraA07g018220.3C*	*RPL10AB*	0.4172	0.0019	0.5375	3.32 × 10^−5^
*BraA07g022360.3C*	*RPS26B*	0.3703	0.0063	0.4319	0.0012
*BraA07g031310.3C*	*RPL17B*	0.3695	0.0065	0.4108	0.0022
*BraA08g004460.3C*	*RPL18*	0.3652	0.0072	0.5671	9.55 × 10^−6^
*BraA09g019250.3C*	*ARP1*	0.2793	0.0428	0.357	0.0087
*BraA09g022110.3C*	*RPL32A*	0.3953	0.0034	0.4913	0.0002

**Table 8:** Correlation between hybrid PGW and parental gene expression levels in terpenoid main chain biosynthesis.

GeneID	Symbol	PGW		MPH of PGW	
		cor	Q Value	cor	Q Value
*BraA01g044250.3C*	*IPP2*	−0.4225	0.0016	−0.3671	0.0069
*BraA02g023510.3C*	*HMG1*	−0.3538	0.0094	−0.3694	0.0065
*BraA02g028120.3C*	*HMGS*	−0.3386	0.0131	−0.5465	2.30× 10^−5^
*BraA03g011760.3C*		−0.353	0.0095	−0.3878	0.0041
*BraA06g027360.3C*	*FLCY*	−0.3564	0.0088	−0.5517	1.85 × 10^−5^
*BraA07g002120.3C*		−0.3555	0.009	−0.4595	0.0005
*BraA08g025620.3C*	*ICMEL1*	−0.391	0.0038	−0.5731	7.30 × 10^−6^
*BraA09g014810.3C*	*ISPF*	−0.3647	0.0073	−0.3582	0.0084

## Data Availability

The RNA-seq data have been deposited with the NCBI with the dataset identifier PRJNA876066.
